# Retraction: Sun L. *et al*. Porosity Defect Remodeling and Tensile Analysis of Cast Steel. *Materials* 2016, *9*, 119, doi:10.3390/ma9020119

**DOI:** 10.3390/ma9060488

**Published:** 2016-06-18

**Authors:** Materials Editorial Office

**Affiliations:** Klybeckstrasse 64, CH-4057 Basel, Switzerland; materials@mdpi.com

We have been made aware that the figures and experimental data of this article [1] are duplicated from another publication by Hardin *et al*. [2]. In addition, the first author Linfeng Sun submitted and published the manuscript without the knowledge of the other authors by fabricating contact email addresses.

MDPI is a member of the Committee on Publication Ethics and takes the responsibility to enforce strict ethical policies and standards very seriously. To ensure the addition of only high quality scientific works to the field of scholarly publication, [1] is retracted and shall be marked accordingly. The article is retracted with the agreement of all authors. We apologize to the readership of *Materials* for any inconvenience caused.
